# Complex Y chromosome anomalies in an infertile male

**DOI:** 10.5935/1518-0557.20200022

**Published:** 2020

**Authors:** Nirmani P Kaluarachchi, Malshani H Randunu, Munshifa Jainulabdeen, Arunthusha Thavarajah, Padmapani Padeniya, Prasanna Galhena

**Affiliations:** 1 Department of Cytogenetics, Lanka Hospitals Diagnostics, Sri Lanka; 2 Faculty of Medicine, University of Kelaniya, Sri Lanka

**Keywords:** male infertility, Y chromosome anomalies, azoospermia, AZF microdeletion

## Abstract

Y chromosome anomalies are closely associated with non-obstructive azoospermia (NOA), a major etiology in male infertility. Klinefelter syndrome (KS) and Y chromosome microdeletions are some of the well-identified genetic defects in this regard, while Y chromosome aneuploidies have been reported to be susceptive. We report the rare case of a patient presenting with three complex genetic defects: mosaic Y chromosome aneuploidy; loss of the heterochromatin region in the q arm of the Y chromosome (Yqh-); and azoospermia factor C subregion (AZFc) microdeletion. The patient reported he had been subfertile for five years. Semen analysis confirmed total azoospermia along with an unaffected hormonal profile for serum follicle stimulating hormone (FSH), luteinizing hormone (LH), and prolactin levels. Since the microdeletion analysis of azoospermia factor (AZF) region revealed the presence of three microdeletions in the AZFc region, the patient was offered intracytoplasmic sperm injection (ICSI) upon the retrieval of sperm by testicular sperm extraction (TESE) as the best possible assisted reproductive treatment (ART) option. It was further suggested to carry out pre-implantation genetic screening (PGS) in order to facilitate the transfer of only female embryos, thus preventing the dissemination of Y chromosomal anomalies.

## INTRODUCTION

Infertility is a global problem in which males account for 20-70% of affected partners. Though etiologies for male factor subfertility are multiple and multifactorial, genetic causes have increasingly been recognized as one of the main causes recently (Tournaye *et al*., 2017). Male infertility is closely associated with Y chromosome anomalies resulting in defective gonadal development and spermatogenesis (Küçükaslan *et al.*, 2013). Non-obstructive azoospermia (NOA) has been defined as “absent sperms in the ejaculate due to failure of spermatogenesis” and thought to be closely associated with genetic anomalies (Fogle *et al.,* 2006). In addition, the higher prevalence of abnormal karyotypes among patients with azoospermia or severe oligospermia justifies the need for extensive genetic profiling of such affected patients (Chiba *et al*., 2016). Management of these patients is entirely based on ART and has very little scope unless they are coupled with ICSI and TESE (Chan *et al.,* 2001). Therefore, male factor subfertility needs to be properly investigated with the objective of fleshing out the exact etiology, so that the best ART option can be offered. This paper reports the case of a patient with non-obstructive azoospermia and three genetic defects: mosaic Y chromosome aneuploidy; Y qh- deletion; and AZFc microdeletion, one of the rarest defects. 

## CASE DESCRIPTION

A 37-year-old male came to the subfertility clinic claiming he had been subfertile for five years. The patient gave a vague account in which he stated he had mumps at the age of 11 and reached spermarche in his early teens. There was no significant family history of subfertility and his own brother was married and had two healthy children. Physical examination findings indicate he was a healthy adult with a body mass index (BMI) of 23.5 kg/m2 without evidence of gynecomastia. He had a normal phallus and bilaterally descended testes graded as Tanner V. There was no evidence of varicocele or hydrocele. The patient underwent detailed hormonal, genetic, and seminal fluid analysis.

### Hormone assays

Complete analysis of hypothalamic-pituitary axis was performed based on serum FSH, LH, and prolactin levels. The patient had a marginally elevated serum FSH level [11.31mIu/ml (0.7-11.1)] along with normal prolactin [367.71mIU/L (70-410)] and LH [4.11mIu/ml] levels.

### Semen analysis

Comprehensive semen analysis carried out twice showed the patient had azoospermia with normal macroscopic appearance and insignificant amount of other cellular components.

### Cytogenetic analysis

**Figure 1:**
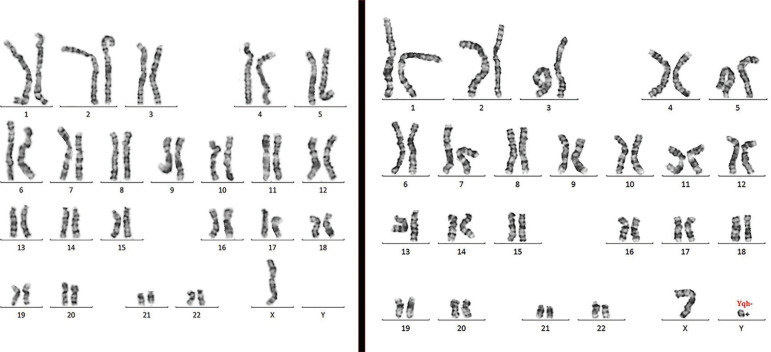
GTG analysis of metaphases showing loss of Y or Monosomy X, i.e. 45,X (left) and a loss of the heterochromatin region in the q arm of the Y chromosome, i.e. 46,X,Yqh- (right).

Peripheral blood lymphocytes were cultured and harvested as per the original protocol described by Moorhead *et al*. (1960). A total of 20 metaphases with GTG banded chromosomes were analyzed and karyotypes were reported in accordance with the guidelines of the International System for Cytogenomic Nomenclature (ISCN) 2016. Chromosome analysis of 20 metaphases from cultured peripheral blood using GTG banding revealed a karyotype of 45,X[7]/46,XYqh-[13] (Figure 1).

### Fluorescence *in situ* hybridization (FISH)

**Figure 2:**
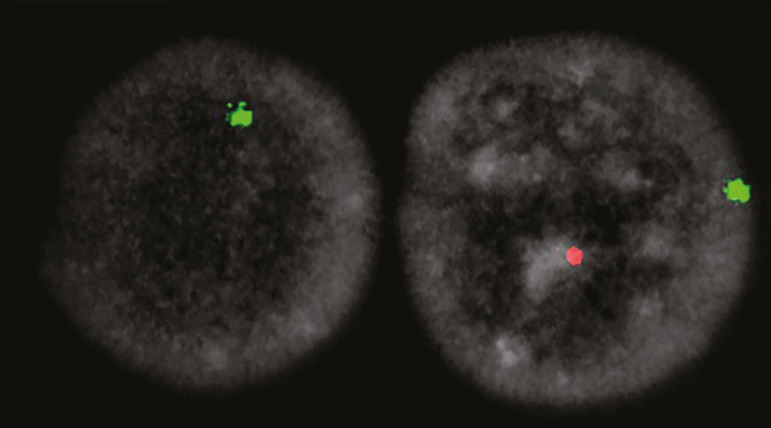
FISH analysis of interphase nuclei with specific centromeric probes for chromosome X (green) and Y (red), showing loss of Y or Monosomy X (left) and a normal signal male pattern (right).

Cultured peripheral lymphocytes were harvested, mounted, and fixed on previously coded slides. FISH was performed using a MetaSystems XCyting Centromere Enumeration Probe for Chromosome X and Y on cultured interphase nuclei. A total number of 200 cells were analyzed by two observers and the presence of Y chromosome mosaicism was confirmed in 35% of the cell population having 45X chromosome complement (Figure 2).

### Detection of AZFa, b, and c subregion microdeletion

Presence of Yqh- in the karyotype drove Y chromosome microdeletion analysis. Molecular genetic analysis was carried out using a multiplex polymerase chain reaction (PCR) method targeting the following sequence-tagged sites (STS): AZFa: sY81, sY84s, Y86, sY182; AZFb: sY114, sY121, sY124, sY127, sY128, sY129, sY130, sY133, sY134, sY143, sYPR3, RBMY1; and AZFc: sY149, sY153, sY157, sY158, sY208, sY242, sY254, sY255, sY145, SY147, sY152. The test found microdeletions in the AZFc subregion (sY254, sY157, sY145) associated with varying phenotypes.

## DISCUSSION

More than 30 million men worldwide are estimated to suffer from infertility with significant genetic predispositions affecting both spermatogenesis and androgenesis (Tournaye *et al*., 2017). Sex chromosome anomalies such as KS, and certain autosomal abnormalities are reportedly important in this regard. In the present case, we observed a karyotype of mosaic Y chromosome aneuploidy along with a polymorphic variant in the long (q) arm of the Y chromosome 45,X[7]/46,X,Yqh-[13]. Results of FISH also confirmed the presence of Y chromosome aneuploidy in 35% of the analyzed cell population. Mosaic Y chromosome aneuploidy 45,X/ 46,XY is a rare chromosomal abnormality which gives rise to varying phenotypic features of gonadal development such as expression of a female phenotype, indefinite genitals, or a male with hypospadias or azoospermia (Chiba *et al*., 2016). The extent of Y chromosome mosaicism observed in this patient gave rise to an apparently normal male phenotype with azoospermia.

The 46,XYqh- karyotype is a common polymorphic variant that occurs in the general population with no significant clinical implications (Gao *et al.,* 2017). Most of these polymorphic variants represent either loss or gain in heterochromatin regions of the same chromosome that is genetically silent. The sex determining gene (SRY) and the AZF-a, b and c subregions, which respectively reside in the p and q arms of chromosome Y, are involved in male gonadal development and spermatogenesis (Liu *et al.,* 2016). Reports have cited loss of the heterochromatin region in the q arm of the Y chromosome and speculated around the involvement of the AZF region, which directed microdeletion analysis to the AZF region.

The AZF region in the q arm of the Y chromosome plays an important role in spermatogenesis and differentiation (Samli *et al*., 2006). The presence of five recombination hotspots in the q arm of the Y chromosome and their unusual molecular arrangement has made this particular region more prone to deletion. Three clinically recognizable submicroscopic deletions have been identified in the AZFa, AZFb, and AZFc subregions (Tournaye *et al*., 2017; Zorrilla & Yatsenko, 2013). Complete or partial deletion of these subregions has been clearly associated with azoospermia or severe oligozoospermia unrelated to the testicular phenotype (Tournaye *et al*., 2017). Identification of these submicroscopic deletions has great diagnostic and prognostic value. Men with complete AZFa, and AZFb deletions do not have spermatozoa during a TESE procedure, whereas men with AZFc deletion - although they have virtually zero spermatozoa in the ejaculate - may have some spermatozoa in testicular tissues (Zhang *et al.,* 2013). The proband in this reported case had AZFc deletion and might be a candidate for in vitro fertilization (IVF) and PGS after TESE (Zorilla & Yatsenko*,* 2013). This would enable the couple to transfer only female embryos rather than an embryo with the 46,XYqh- anomaly, thus preventing the transmission of the Y chromosome microdeletion. Therefore, AZF microdeletion analysis should be carried out when a loss of q arm heterochromatin region in Y chromosome is detected.

Therefore, genetic testing is mandatory whenever a patient presents with azoospermia. Baseline karyotyping is essential in order to rule out sex chromosome aneuploidies. Understanding the nature of Y chromosome microdeletions may optimize patient management and enable the choice of the best possible method of ART.
